# Humidity Sensing Applications of Lead-Free Halide Perovskite Nanomaterials

**DOI:** 10.3390/ma15124146

**Published:** 2022-06-10

**Authors:** Kevin Tambwe, Natasha Ross, Priscilla Baker, Thanh-Tuân Bui, Fabrice Goubard

**Affiliations:** 1 SensorLab, Chemical Science Building, University of the Western Cape, Robert Sobukwe Drive, Bellville, Cape Town 7535, South Africa; nross@uwc.ac.za; 2 CY Cergy Paris Université, LPPI, F-95000 Cergy, France; tbui@cyu.fr

**Keywords:** halide perovskite, lead-free, nanomaterials, humidity sensors, capacitive/resistive sensor, fabrication technologies, real-time application

## Abstract

Over the past decade, perovskite-based nanomaterials have gained notoriety within the scientific community and have been used for a variety of viable applications. The unique structural properties of these materials, namely good direct bandgap, low density of defects, large absorption coefficient, high sensitivity, long charge carrier lifetime, good selectivity, acceptable stability at room temperature, and good diffusion length have prompted researchers to explore their potential applications in photovoltaics, light-emitting devices, transistors, sensors, and other areas. Perovskite-based devices have shown very excellent sensing performances to numerous chemical and biological compounds in both solid and liquid mediums. When used in sensing devices, Perovskite nanomaterials are for the most part able to detect O_2_, NO_2_, CO_2_, H_2_O, and other smaller molecules. This review article looks at the use of lead-free halide perovskite materials for humidity sensing. A complete description of the underlying mechanisms and charge transport characteristics that are necessary for a thorough comprehension of the sensing performance will be provided. An overview of considerations and potential recommendations for the creation of new lead-free perovskite nanostructure-based sensors is presented.

## 1. Introduction

The active materials of humidity sensors can be categorized into metal oxides, with perovskites being a subpart [1], carbon materials [2], and polymer composite [3]. Among metal oxides, compounds such as ZnO [4], Al_2_O_3_ [5], and In_2_O_3_ [6] have been the most commonly used materials for humidity sensors, and they also fall within a class of compounds named ceramics. The high thermal, chemical, and mechanical stability of these ceramic compounds makes them suitable for humidity sensing applications. Additionally, a recent study demonstrated that the introduction of conductive indium tin oxide and dielectric aluminum oxide inks allows for a larger sensing range of humidity (5–95%) and the potential of increasing the detection surface area [6]. Regarding carbon materials, Graphene oxide (GO) and nanofibrillated cellulose (NFC) have also been extensively used in humidity sensing applications. The advantages that GO and NFC offer are that they both have a rather large specific area with hydrophilic groups (such as hydroxyl (-OH) and carboxyl(-COOH)) allowing the presence of a significant number of active sites for the absorption of water molecules. Despite this, the interesting physical properties offered by both metal oxides and carbon materials do not make up for the inherent brittleness and potential undesired morphological structures (inhomogeneous dispersal of pores with inadequate porosity supplements). A possible way to deal with these physical anomalies is through the addition of elastomers and polymers to help enhance their flexibility [7,8]. On the other hand, polymers used in humidity sensors offer the advantage of having a controlled structural formation, a simple solution processing, and can be prepared in batches; they show great potential in medicine, notably for tissue engineering [9] or respiration monitoring [10].

In recent years, lead halide perovskites with a general formula of APbX_3_ (with A = Cs, Rb, {CH_3_NH_3_}^+^(MA) or {HC_9_NH_2_)_2_}^+^(FA) and X = Cl, Br or I) have experienced an exponential rise in their use as multifunctional materials with considerable attention placed on their optoelectronic applications [11]. Due to their amazing optical absorption, low exciton binding energy, low trap state density, extended charge carrier lifetime, and tunable bandgap, lead halide perovskites have had significant success as active materials in devices such as solar cells [12,13,14,15]. However, despite their remarkable properties, performance, and various applications, there have been concerns over the toxicity and intrinsic instability of lead halide perovskite materials [16,17]. The toxic lead content of the various devices made from lead halide perovskites constitutes the key concern that hinders their large-scale commercialization. An additional major issue is the chemical instability of the lead halide perovskite structure (i.e., they are prone to decomposition into their original precursors) when exposed to external factors such as heat, oxygen, moisture, and even light illumination [18]. Hence, there is a need for the development of new mitigation strategies for designing lead-free, stable, and non-toxic perovskite materials.

A certain number of studies looking to remediate the issue of lead toxicity have been done by researchers over the past few years. One way in which they were able to minimize the incidence of lead poisoning or its presence was through the replacement of Pb atoms with other suitable non-toxic, low-cost, and environmentally friendly divalent, trivalent, or tetravalent metal cations (such as Sn^2+^, Ge^2+^, Sb^3+^ or Bi^3+^) [19,20,21,22,23,24]. However, while lead-free perovskites have yet to achieve high competitive power conversion efficiencies in solar cells, they on the other hand do show promise as a transducer material in sensor systems [25].

Lead-free halide perovskites have also been chosen as possible material for use in humidity sensors; this is one of their much lesser-known application, hence the need to investigate these materials to further exploit their very promising capabilities in this field. The environmental friendliness, stability, and adjustable optoelectronic capabilities of lead-free double perovskite materials have made these types of perovskites very attractive within the research community. Among them, Cs_2_AgBiBr_6_ is a double perovskite with exceptional optoelectronic characteristics and higher environmental stability. Most double perovskites have greater decomposition energies and stability than equivalent lead-based hybrid perovskites, particularly with Ag-Bi. When compared to ABX_3_ perovskites, they have a much larger excitation binding energy [26]. Humidity sensors with lead-free perovskites as the sensing material are starting to draw a tremendous amount of interest, primarily owing to their various possible applications in environmental control and industrial processing [27]. Their utilization could potentially be more frequent and diverse, especially in intelligent systems used to regulate the weather forecast and measure soil moisture levels during irrigation, and in medical instruments or civil engineering [27]. Various studies were done on lead-free halide perovskite materials and their mechanisms for use in humidity sensors [28,29,30,31]. These studies focused on developing humidity sensing materials with high sensitivity, short response/recovery time, great linearity, and minimal hysteresis. Parameters such as sensitivity and response/recovery times are the key parameters to consider when it comes to humidity sensing device evaluation and research as a whole [32,33]. However, these sensors should maintain their long-term stability and durability, especially in high-humidity conditions.

## 2. Lead-Free Halide Perovskites: Structure, Stability, and Characteristics

From the traditional structure of cubic symmetry, perovskites present a multitude of crystalline variations [34], all depending on their composition, stoichiometry, and synthetic conditions. Perovskites ceramics such as PbTiO_3_, CaTiO_3_, ZnSnO_3_, and NaTaO_3_ have proven to be interesting materials for humidity sensing applications owing to their chemical, thermal stability, and high sensitivity [27,35]. Their response and recovery times, on the other hand, are significantly longer (generally 10 s or more), which limits their application in real-time humidity monitoring [36]. Particularly in the medical field, where monitoring one’s breathing, for example, necessitates the application of short response and recovery humidity sensors (e.g.: sensors in medical ventilators which help convey some warm air and wet air). Among perovskite materials, lead-free halide perovskites have also shown very promising humidity-sensing capabilities. State of the art studies demonstrated that two main structures are good candidates for humidity sensor application: 3D structure-based lead-free halide perovskites such as Cs_2_BX_6_ (B = Ag, Bi, Sn, Te, Ti, Pd and X = Br, Cl or I) [37] or Cs_2_B’B” X_6_ (B′ = Sb^3+^, Bi^3+^; B″ = Cu^+^, Ag^+,^ Au^+^ and X2= Br, Cl or I)) [38] and low-dimensional based perovskites such as A_2_InX_5_.H_2_O (A = K, Rb, Cs, and X = Br, Cl or I) [39] have shown very promising properties which have sparked significant research interest. 

### 2.1. Vacancy Ordered and Double Perovskite Compounds

The typical cesium double perovskites (Cs_2_B’B”X_6_) [40,41,42,43,44,45] and the vacancy-ordered cesium double perovskites (Cs_2_BX_6_) [46,47,48,49,50] have also been successfully prepared via either a simple Pb^2+^ substitution from CsPbX_3_ with paired monovalent and trivalent metal cations or Pb^2+^ substitution by tetravalent cations and one vacancy site. This newly found perovskite family which has the chemical formula Cs_2_B′B″X_6_, (B′ = Sb^3+^, Bi^3+^; B″ = Cu^+^, Ag^+^, Au^+^) may give a potential range of materials for sensors that are both environmentally benign and stable [38] as well as other optoelectronic applications [51,52,53,54]. Hence, halide perovskites from the Cs_2_BX_6_ (or Cs_2_BX_2′_X_4_) and Cs_2_B′B″X_6_ family containing less toxic metals have been proposed notably with Cs_2_AgBiBr_6_, Cs_2_BiAgCl_6_, Cs_2_SnI_6_, Cs_2_TeI_6_, Cs_2_TiBr_6_, Cs_2_PdBr_6_, etc. [47,51,52,53,54,55,56].

**Figure 1:**
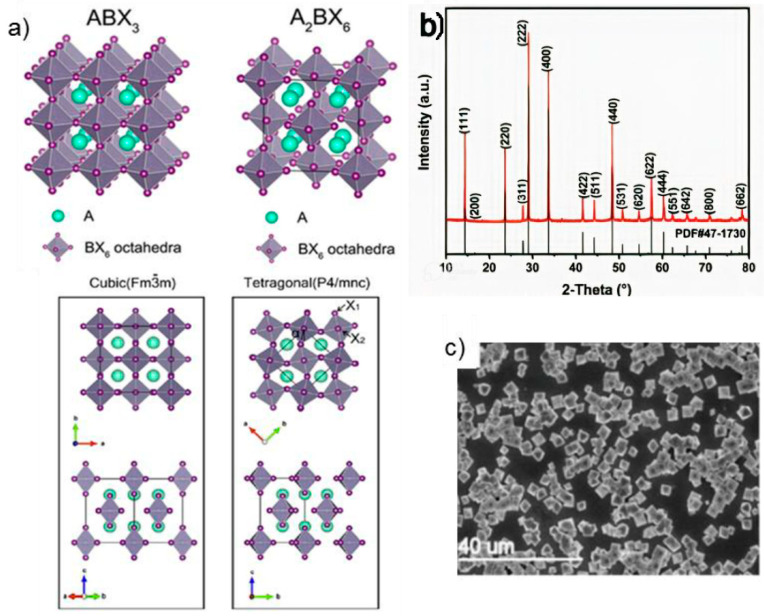
(**a**) Crystal structures of ABX_3_ (top left) and A_2_BX_6_ (top right), and A_2_BX_6_ compound in cubic phase (down left) and tetragonal phase (down right) of polymorphs viewed from various angles. (**b**) XRD pattern of Cs2PdBr6 powders. (**c**) SEM image of Cs_2_PdBr_6_ powders. Reprinted/adapted with permission from Ref. [67]. Copyright ©2022, American Chemical Society. and Ref. [37]. Copyright ©2020, Royal Society of Chemistry.

The lead halide perovskite (APbX_3_) typically has a three-dimensional network of corner-sharing [PbX_6_]_4_ octahedra, with Pb^2+^ ions in the core and A^+^ ions occupying the wide cavity between neighboring octahedra (Figure 1a) [57]. A heterovalent substitution is used to replace the toxic lead atoms while keeping the three-dimensional perovskite connectivity and its advantageous optoelectronic characteristics [58]. As indicated in Figure 1a, the unit cell would normally be duplicated, and a pair of lead ions (Pb^2+^) is substituted by combining one monovalent (B^+^) and one trivalent (B^3+^) ion (b). Double perovskite is the resultant substance, having the formula A_2_B’B”X_6_ and the cubic space group Fm^3^ m [59]. The geometrical tolerance factor (t) and specific octahedral factor (μ) are key parameters to be taken into account while designing stable double perovskites [60]. The theoretical parameters for a stable double perovskite structure are μ > 0.41 and 0.75 < t < 1.017, respectively. The ion radius is also essential in the construction of a double perovskite. The octahedral factor prevents bulkier anions, such as iodide, from being used in Cs_2_AgBiX_6_. With Cl and Br in Cs_2_AgBiX_6_, being the only double perovskites structures that can be formed [58]. Lead-free double perovskites possess several favorable properties, including low defects, excellent optical absorption, suitable bandgap, and ultra-high stability.

Many candidates for this design have been synthesized successfully, including Cs_2_BiAgCl_6_, Cs_2_BiAgBr_6_, Cs_2_SbAgCl_6_, and Cs_2_SbAgBr_6_ [61]. Because of its exceptional photoelectric characteristics, Cs_2_BiAgBr_6_ has been hailed as a potential semiconducting material [62]. Cs_2_BiAgBr_6_ has a bandgap of 2.19 eV [54] and a carrier mobility of 0.3 cm^2^ V^−1^s^−1^ [63], indicating that it is a potential lead-free optoelectronics material. As a result, it was initially developed into a lead-free halide double perovskite solar cell with a PCE of more than 2.5 percent [64], as well as a stunning performance as an X-ray detector [65]. The excellent environmental stability of Cs_2_BiAgBr_6_ is one of its unique characteristics [66]. Furthermore, given the exceptional humidity-dependent electrical characteristics of Cs_2_BiAgBr_6_, the lead-free Cs_2_BiAgBr_6_ perovskites’ potential as a humidity sensor should be studied even further. 

Cs_2_BX_6_ compounds on the other hand have proven to have enhanced stability in ambient environments, correlating with their ability to host a metal such as Sn, Ge, etc., in their 4+ oxidation state which is much more stable. The BX_6_ octahedra of the Cs_2_BX_6_ structure are also found to be isolated, in contrast to the corner-sharing arrangement that defines the CsBX_3_ perovskite structure (Figure 1a, top). The B-X bond lengths in the Cs_2_BX_6_ molecule are shorter as a result of this structural shift, which has been linked to its increased chemical stability. Figure 1a—down) depicts the cubic and tetragonal crystal structures of A_2_BX_6_ (in this case Cs_2_BX_6_) compounds. Among the various Cs_2_BX_6_ compounds, Cs_2_SnI_6_ recently discovered by Snaith et al. showed that in general compounds A_2_BX_6_ exhibit long-lived photoluminescence with an optical bandgap of about 1.6 eV and are extremely moisture resistant [68]. 

Cs_2_PdBr_6_ and other members of the Cs_2_BX_6_ perovskite family are molecular salts rather than true perovskites. Cs_2_PdBr_6_ is a soluble perovskite that eliminates many of the limitations of the CsBX_3_ type of perovskite. Compounds such as Cs_2_PdBr_6_ possess a cubic shape (Figure 1b,c) with great symmetry and exceptional air and moisture stability. Aside from the Pb substitution, the presence of non-toxic metals in these types of compounds also solves the environmental instability of perovskites. Cs_2_PdBr_6_ was used as a humidity sensor by Ye et al. for the detection of fruit waxing [37].

For the synthesis of Cs_2_B’B”X_6_ Compounds such as Cs_2_BiAgBr_6_ [38], it is done via the temperature lowering crystallization method. This technique involves the dissolution of solid BiBr_3_ and CsBr (1:2 mol %) into 12 mL of 48% HBr. The mixture is heated to 140 °C and stirred on an oil bath magnetic mixer to speed up the dissolution process. Solid AgBr (1 mol %) is then added to the solution. After that, the mixture is kept stirring on the oil bath magnetic mixer to dissolve all the residual precipitation. The solution is held at 140 °C for 3 h, and then very slowly cooled to room temperature by 2 °C per hour. The red crystals are obtained after about two days. Larger crystals are obtained by controlling the cooling rate at 1 °C per hour. The synthesis of Cs_2_BX_6_ compounds such as Cs_2_PdBr_6_ [37] on the other hand, is done via the preparation of a mixture of CsBr and PdBr_2_ (2:1 mol %) dissolved in a mixture of HBr and DMSO. After the heating and stirring process, the precipitated Cs_2_PdBr_6_ is filtered, washed, and left to dry, resulting in a high-quality crystalline powder (see Figure 1c) and octahedral microcrystals with a 3 µm diameter. This perovskite material is highly resistant to moisture, light, and heat which allows them to exhibit good long-term structural stability. 

### 2.2. A_2_InX_5_∙H_2_O Perovskite Compounds

The low dimensional indium-based perovskite, which is considered to have superior luminous capabilities in terms of quantum confinement, is another interesting prospect for humidity sensing applications. However, very little research on the synthesis of the A_2_InX_5_·H_2_O (A = K, Rb, Cs) has been published [69,70].

**Figure 2:**
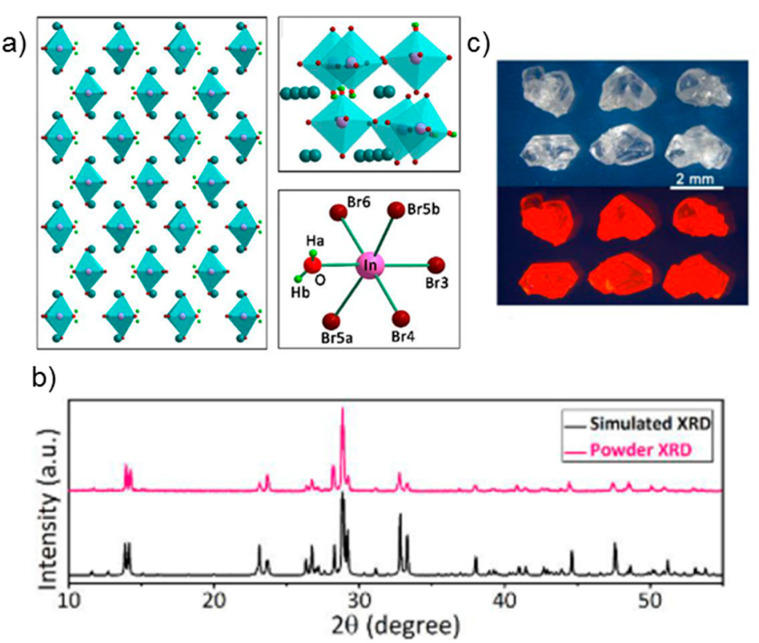
(**a**) Crystal structure of Cs_2_InBr_5_·H_2_O viewed along the (001) axis (cyan: Cs, brown: Br, pink: In, red: O, green: H). (**b**) SCXRD and PXRD patterns of Cs_2_InBr_5_·H_2_O. (**c**) Images of Cs_2_InBr_5_·H_2_O single crystal under ambient light (up) and UV light (bottom). Reprinted/adapted with permission from Ref. [39]. Copyright © 2022 Wiley-VCH Verlag GmbH & Co. KGaA, Weinheim.

Taking the example of Cs_2_InBr_5_·H_2_O which is one of the most prevalent members of the A_2_InX_5_·H_2_O perovskite family of materials, the single crystals of Cs_2_InBr_5_·H_2_O are prepared by temperature-lowering crystallization method [39]. The single crystal obtained from this method adopts an orthorhombic type of configuration and is isomorphous. The In, O, and three Br atoms are positioned on the mirror plane. Figure 2a,b give a detailed overview of the crystal structure of Cs_2_InBr_5_·H_2_O, in which the presence of Cs^+^ allows for the InBr_5_O octahedron to be spatially isolated and form a 0D structure. A very important aspect of this structure is that to completely exclude any form of interactions between the InBr_5_O octahedrons, the two adjacent In ions are separated by a distance of 7.1 Å. As opposed to the conventional PbBr_6_^4−^ lead halide octahedron which is made up of the same type of anions, the InBr_5_O octahedron is composed of different atoms, that is to say, five Br atoms along with an O atom coming from the H_2_O as seen in Figure 2c. The InBr_5_O octahedron is structurally stabilized by the significant coordinating effect O and In have.

Additionally, these indium-based materials have been found to have coupled water processes with reversible release/uptake and as a result of the switchable dual emission, they make excellent PL water sensors in humidity and organic solvents (Figure 2c). They are characterized by a unique 0D structure and exhibit broad emission (~695 nm) with a high PLQY of 33%. These luminescent lead-free perovskite bulk materials may help pave the way for metal halide perovskite to be used in water detection.

Novel A_2_InX_5_.H_2_O perovskite molecules such as Cs_2_InBr_5_·H_2_O are synthesized via the temperature-lowering crystallization method [39]. This method allows for the slow formation of the Cs_2_InBr_5_·H_2_O perovskite crystals, that are of the orthorhombic type of configuration. As can be seen from the XRD pattern of the single crystal in Figure 2b, the single-crystal data correlates with the powder X-ray diffraction (PXRD) pattern. 

### 2.3. Factors That Make Lead-Free Inorganic Perovskite Materials Suitable for Humidity Sensing

**Figure 3:**
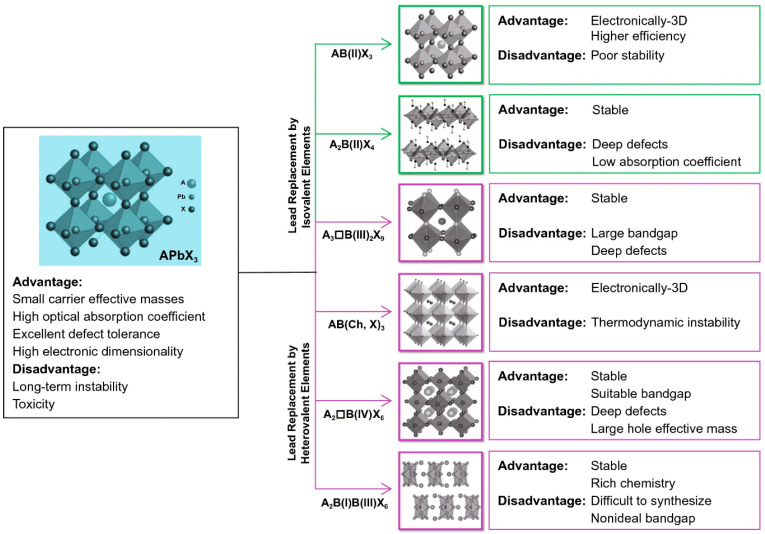
Schematic illustration of the approaches and consequences of potential Pb replacement. bottom). Reprinted/adapted with permission from Ref. [72]. Copyright © 2022 Springer Nature Switzerland AG. Part of Springer Nature.

After several years of focused investigation, researchers have gained a good grasp of the lead-free perovskite system. The substitution of the Pb element lies at the heart of research toward lead-free perovskite materials. Through theoretical calculations, researchers have ruled out several elements that can potentially replace Pb (Figure 3). Stability and desired bandgap were two concurrent conditions for good candidates for lead-free perovskites, according to Filip et al. [71]. They investigated the stability of potential lead-free perovskites using randomly shifted structures (atom locations and lattice characteristics). The crystal structure keeps the perovskite geometry following the relaxation of the disrupted configurations, and the relativistic bandgap is less than 2.0 eV, making the stability and required predicted bandgap two concurrent criteria. Cu, Ag, Bi, and Sb have proven to be good enough to replace Pb in the formation of perovskites in later studies [54,62,63,64,65]. Using the first-principles computation, it has been demonstrated that it is also feasible to employ valence state substitution to choose acceptable non-toxic elements [72]. We may usually substitute the Pb element with homovalent elements like Sn, Ge, and Cu, or heterovalent elements like Sb and Bi, as illustrated in Figure 3. The heterovalent replacement can be separated into three subcategories to preserve charge neutrality, namely cation splitting, mixed-valence anion, and ordered vacancy [72]. However, among these three sub-categories, mixed-valence anion (double perovskites) and vacancy ordered perovskite have proven to be the most stable.

By dividing Pb into a mixture of monovalent and trivalent cations, a double perovskite structure with the chemical formula A_2_B(I)B(III)X_3_ is formed. When a single valence cation but two valence anions are introduced, the general chemical formula becomes AB(Ch, X)_3_, where Ch represents a chalcogen element and X represents a halogen element. By creating ordered vacancies, the perovskite may retain electrical neutrality. These substitutions are further classified into two types: B(III) compounds with the formula A_3_B(III)X_9_ and B(IV) compounds with the formula A_2_□B(IV)X_6_. Vacancies are indicated by the symbol □ in this case. The creation of vacancies, on the other hand, transforms the original 3D perovskite structure into a low-dimensional crystal structure, lowering the electrical dimension and thereby affecting optoelectronic performance [73].

Previous research has shown that tetravalent B(IV) replacements may be used to replace Pb in lead halide perovskites. To accommodate these heterovalent replacements, the chemical formula must be changed to A_2_BX_6_, which is obtained by removing half of the B-site cations from the ABX_3_ perovskite structure. The A_2_BX_6_ perovskite form is frequently referred to as the A_2_B□X_6_-type vacancy ordered double perovskite because of the huge charge difference between them [67,74,75,76]. In the A_2_BX_6_ perovskite structure, the B-site vacancies (designated as **□**) and the remaining B-site cations are arranged in a rock salt pattern. The A_2_BX_6_ perovskite variations are essentially 0D non-perovskites due to the lack of connection between the [BX_6_] octahedra, even though researchers would want to name them, perovskites. Due to the isolated [BX_6_] octahedra in A_2_BX_6_ compounds, the optoelectronic properties of A_2_BX_6_-type perovskites deviate from those of 3D ABX_3_ (B = Pb, Sn, and Ge) perovskites. A_2_SnI_6_ (A = Cs, MA) [77,78] and Cs_2_TiBr_6_ [76,77] have been explored for photovoltaic applications among the A_2_BX_6_ compounds [47,77,78,79].

Cs_2_BiAgBr_6_ and Cs_2_PdBr_6_ are both recently developed lead-free perovskite with good photoelectric properties and high stability. Zhan Yiqiang et al. and Ye et al. both found that these materials reported humidity-dependent electrical properties and good stability and therefore giving them excellent humidity sensitivity [37,38]. By analyzing the humidity response of the material at different temperatures and relative humidity levels, they were able to establish that the adsorption effect of water molecules on the surface of these perovskites is the main cause of the humidity-sensitive mechanism.

## 3. Applications

### 3.1. Humidity measurement

There are two types of humidity measurements, i.e., absolute humidity, and relative humidity related to qualitative assessment. The proportion of vapor (moisture) in a unit volume of air, independent of temperature, is referred to as absolute humidity. The proportion of water vapor present in a unit volume of air is also referred to as relative humidity, but this time in connection to the temperature of the air.

Most humidity sensors work by measuring the change in capacitance or resistance of a certain conductive substance as a function of relative humidity. Several fundamental parameters determine the good functioning of a humidity sensor. The proper operation of a humidity sensor is determined by several essential parameters. The key parameters to consider when it comes to evaluating the functioning of a humidity sensor are sensitivity, accuracy, and response/recovery time.

### 3.2. Classification of Humidity Sensors

Humidity sensors are categorized into several classes of sensors. These groups include electrical, mechanical, optical, and integrated sensors. Electrical sensors are types of sensors whose functioning is based on impedance and capacitance (Table 1), strain and mass-loading effects are at the heart of mechanical sensors’ operation. One of the more common types of sensors is an optical sensor, which works by transmitting, reflecting, and quenching electromagnetic waves. Lastly, integrated types of sensors comprise electronic parts for linearization, calibration, transmission, etc. [80]. There are, however, many drawbacks to the use of electrical and mechanical sensors. Distance between sensor and signaling circuit for example is a considerable issue that hinders the functioning of capacitive sensors. Exposure to certain external factors such as chemical vapors, oil mist, etc., reduces the long-term stability of resistive sensors and very often causes premature failure. These types of sensors tend to malfunction in the presence of water-soluble coatings. Gases such as nitrogen, which have thermal properties, may disrupt the functioning of thermal conductivity humidity sensors and therefore, negatively impact the selectivity of the sensor [81].

### 3.3. Humidity Sensing Mechanism of Perovskites

**Figure 4:**
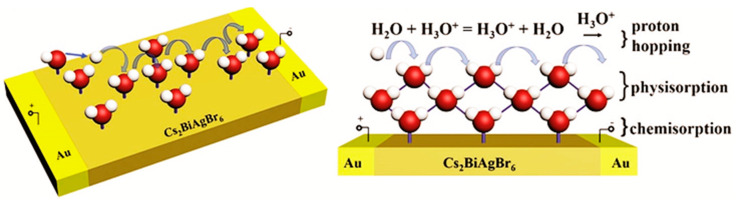
A diagram of the humidity sensing mechanism of Cs_2_BiAgBr_6_ lead-free perovskite thin film. Reprinted/adapted with permission from Ref. [38]. Copyright © 2022 WILEY-VCH Verlag GmbH & Co. KGaA, Weinheim.

The general humidity sensing mechanism of ceramic oxides or perovskites as a whole relies on the superficial water vapor absorption from chemisorption, physisorption, and capillary condensation process as can be seen in Figure 4 [5,38]. As a result of the water absorption occurring on the sensing material surface, electric properties change and affect, consequently, the resistance or capacitance of the sensing material. 

In general, the adsorption of water molecules on the sensor surface during the humidity sensing process is divided into different phases, chemisorption, and physisorption [82,83], as illustrated in Figure 4 with the example of the Cs_2_BiAgBr_6_ humidity sensor. When there are no water molecules adsorbed on the film surface, the conductivity comes from the carriers inside the *p*-type Cs_2_BiAgBr_6_ material, which may principally be the Ag vacancy [62]. Water molecules are adsorbed by chemisorption at low humidity [84]. Meanwhile, according to Anderson and Parks’ proton conductivity model [84,85], in addition to the water molecules chemisorbed in low RH areas, protons are released as a result of the dissociation of water molecules. When the dissociated bare H^+^ protons are transported to nearby water molecules, H_3_O^+^ are generated. In the presence of external electric fields, the protons can then be moved from one site to another across energy barriers. As a result, when the humidity level is low, the sensor’s electrical conductivity rises.

When exposed to a higher RH environment, water molecules can create several physisorbed layers on the surface of the Cs_2_BiAgBr_6_ thin film (see Figure 4) [86]. Many water molecules, in particular, have the potential to bind to form a liquid-like network of hydrogen-bonded water molecule layers. In liquid water, hydration of H_3_O^+^ is energetically favorable. The proton-transfer process follows the formula H_2_O + H_3_O^+^ = H_3_O^+^ + H_2_O, the proton transfer between neighboring H_2_O molecules inside a continuous layer of water, as stated by the Grotthuss ion transfer mechanism [85]. As a result of the free mobility of protons, the resistance of the Cs_2_BiAgBr_6_ thin film humidity sensor decreases significantly.

Finally, with such water multilayer (chemisorbed and physisorbed) on the flat (or porous) surface, these different processes can be involved in explaining the resistance or capacitance changes. Under applied voltage, singly bonded water vapor molecules, for example, become mobile and capable of forming dipoles and electrolyte layers, leading to an enhanced dielectric constant and bulk conductivity. As a result, the surface water protonation, as well as protonic conduction mechanisms, might cause a minor fluctuation in conductivity with humidity adsorption.

In terms of shape, dense and planar surfaces allow water molecules to volatilize during the desorption process, allowing for speedy recovery periods. Porous surfaces of humidity sensing materials, on the other hand, trap condensed water molecules in pores, decelerating the evaporation process and resulting in substantially longer recovery durations than response times [87]. The superior performance of Cs_2_AgBiX_6_ and Cs_2_InBr_5_H_2_O (Table 2), in particular, may be explained by the weaker atomic connection between the surface of these perovskite and water molecules as compared to metal-oxide-ceramic humidity sensors, since the hydrogen bond H-X is weaker than the H-O bond. As a result, the initial layer of water molecules on the perovskite surface can readily volatilize.

### 3.4. Sensing Applications of Lead-Free Halide Perovskites

One of the most important ways in which scientists have been able to improve not just the structural characteristics of perovskite materials, but also their optical characteristics, has been to modify their compositional structure. These modifications have proven to have an impact on the material’s performance and stability in device applications. The capacity that perovskite materials have, to tune their bandgap by using mixed-cation or mixed-halide compositions, enables their optical absorption to be expanded spanning a broader spectrum of wavelengths [88,89]. The Goldschmidt-factor of perovskite crystals (ABX_3_) in their stable state ranges from t = 0.8 to t = 1 [60,90,91]. When left in the ABX_3_ configuration, inorganic perovskites based on Cesium exhibit low t-factor values, indicating poor phase stability. As can be observed from Table 2, a variety of metal oxide ceramics, polymers, and organic-inorganic and Cs-based inorganic halide perovskites that have exhibited improved device performance and structural stability in humidity sensors will be reviewed in this section of the work.

**Table 2:** Metal oxides, organic polymers, oxygenated salt, and halide perovskite among which are used as sensing materials in resistive and capacitive humidity sensors.

Compounds	Sensor Type	Coating Method	Morphology	Humidity Range (%)	Response and Recovery Time (t_res/trec_) (s)	Reference
ITO/alumina (4 cm^2^)	Capacitive	Screen-printing	Thin film	11–95	21.4/4.8	[6]
LiCl/ZnO	Capacitive	Screen-printing	Thin film	11–95	3/6	[92]
GO	Capacitive	Sputtering	Thin film	11–97	15/2.5	[93]
NFC/GO/PDMS ^1^	Capacitive	Drop-coating Freeze drying	Thin Film	11–97	57/2	[8]
CaTiO_3_	Capacitive	Solid-state step sintering	NPs	33–95	14.5/34.27	[94]
PMDS/PPDS ^2^	Resistive	Drop casting	Thin film	33–95	0.29/0.47	[10]
PSDA-b-PEG ^3^	Resistive	Electropolymerization	Thin film	0–95	120/180	[95]
CsPbBr_3_	Resistive	Dip-coating	Thin film	30–95	2 or 3/not measured	[96]
PbTiO_3_	Resistive	Screen-printing	NPs	80–95	------------	[97]
ZnSnO_3_	Resistive	Spin-coating	Thin film	11–97	7/16	[98]
NaTaO_3_	Resistive	Doctor-blading	Thin film	33–95	3/32	[99]
Cs_2_PdBr_6_	Resistive	Wet method	Single crystals	11–95	0.7/1.7	[37]
CH_3_NH_3_PbI_0.2_Cl_2.8_	Resistive	Dip-coating	Thin film	30–90	24/24	[100]
Cs_2_BiAgBr_6_	Resistive	Spin-coating	Thin film	5–75	1.78/0.45	[38]
Cs_2_InBr_5_·H_2_O	PL	crystallization method	Single crystals	30–80	30/not measured	[39]

^1^ NFC: Nanofibrillated cellulose; ^1^ PDMS: Polydimethylsiloxane; ^2^ PMDS: Poly(mercaptopropyl polyhedral oligomeric silsesquioxane-1,4-divinylbenzene-sodium p-styrene sulfonate hydrate); ^2^ PPDS: Poly(pentaerythritol tetra(3-mercaptopropionate)-1,4-divinylbenzene-sodium p-styrene sulfonate hydrate); ^3^ PSDA-b-PEG: a diblock copolymer consisting of poly (diphenylamine sulfonic acid) and poly (ethylene glycol).

Humidity sensors typically use two methods to measure humidity. One of those is resistive sensing, in which water molecules affect the bulk material’s resistance due to chemisorption or physisorption. The second is capacitive sensing, in which the sensor’s capacitance changes when it interacts with water vapor [101]. The type of material utilized to make the sensor usually determines the sensing mechanism; as a result, selecting an appropriate material is critical. After exposure to the numerous chemical species anticipated to be present in the ambient, the material to be utilized for studies should have good sensitivity throughout the whole range of RH with characteristics that are stable over time and heat cycling. Humidity sensors are generally made from three different types of materials. Ceramic metal oxides are the first type, with semiconducting perovskite structures being a prominent variant [102]. Another common material that monitors humidity is organic polymer sheets, which change the impedance of a conducting polymer or function into a dielectric for capacitive sensors [27]. Finally, porous inorganic/organic materials have also proven to be highly effective over the years [103,104,105,106,107,108].

The various perovskite materials utilized in the production of the capacitive and resistive sensors reported in Table 2 were synthesized and deposited in diverse ways. Solid state-step sintering, hot injection, solid-state reaction, hydrothermal technique, basic precipitation reaction, spin-coating, and temperature lowering crystallization are some of the synthetic methods used. The bonding and densification of particles by the application of heat below a material’s melting point is known as solid-state sintering [109]. The hot injection method involves the injection of a cold stock solution containing the precursors into a hot solution comprising a surfactant and a high-boiling point sol-vent [110]. Chemical decomposition processes, in which a combination of solid reactants is heated to form a new solid composition and gases [111], are used in the solid-state reaction method. The hydrothermal method entails heating and pressurizing an aqueous solution as a reaction system in a particular closed reaction vessel to establish a high-temperature, high-pressure reaction environment [112]. When dissolved chemicals react to generate one (or more) solid products, this is known as a precipitation reaction. Spin coating is a technique for depositing thin, homogeneous coatings onto flat surfaces [113]. Temperature lowering crystallization is a method in which crystal nucleation and development are kinetically limited since crystallization is thermodynamically favored at low temperatures [114]. The reported results from the lead-free perovskite-based humidity sensors prepared using the previously mentioned techniques demonstrated significantly lower values of response and recovery times than those of existing conventional sensors. The sensor demonstrated highly reliable, stable, and reproducible results in humidity ranges going from 5 to 97% humidity. Based on these factors alone, it could be concluded that these sensors, with further development, could be extremely promising for the creation of a viable humidity sensor.

#### 3.4.1. Resistive Type Humidity Sensors

**Figure 5:**
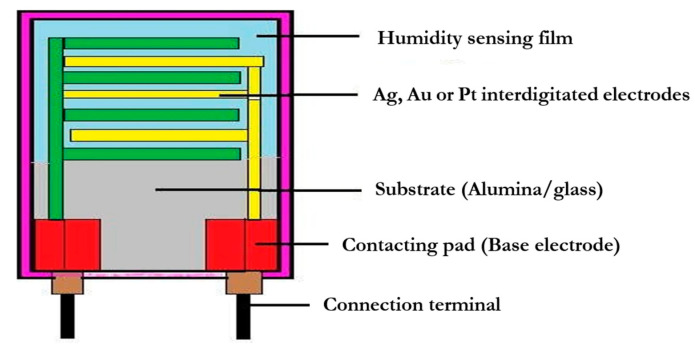
Configuration of a resistive humidity sensor.

Typically, noble precious metal electrodes are fabricated on a glass or ceramic substrate via thick film printing techniques. Ref. [115] or thin-film deposition [116]. Most resistive sensors are designed with interdigitated (interdigital) electrodes [117], with humidity-sensitive films put between them so that the two electrodes are in contact. Electrolytic conductive polymers like salts and acids can be used to coat the platform substrate [118,119], doped ceramic, or perovskites sensing films [35,39,92,120]. Film-based sensors are sometimes constructed by using printing techniques including screen or inkjet printing together with coating techniques like chemical vapor deposition (CVD) methods, spin coating, dip coating, vacuum physical vapor deposition (PVD) techniques, or thermal evaporation and cold sputtering [121]. In hybrid systems, the thick film formed layer is usually the bottom layer. Electrochemical deposition is perhaps the most extensively employed deposition method when a small area has to be covered with prepared polymers. Nonetheless, different deposition techniques, such as spray approaches, are used in a few studies [122] or a combination of spray pyrolysis and other methods [123]. Figure 5 shows an experimental schematic of a planar thick/thin film humidity sensor based on an interdigitated structure with a porous membrane, with some of the key design features highlighted.

The key active materials used for the fabrication of humidity sensors include metal oxides, with perovskites being a subpart [1], carbon materials [2], and polymer composite [3].

##### Polymer-Based Resistive Humidity Sensors

Studies conducted on polymeric humidity sensors have progressed and been incorporated into the industry over the past four decades. The majority of these sensors are made from porous polymer thin films [124] and employ metal oxide ceramic sensors as a model for detecting. The functioning of the sensors is based on the physical and chemical water absorption of the films, as well as condensation in the presence of capillary holes, which causes a change to the physical and electrical properties of the transducer. Structural characteristics dictate the extent of variations in bulk conductivity and dielectric permittivity.

Organic polymer thin film humidity sensors with their applications, on the other hand, have a lower degree of satisfaction and significance when compared to metal-oxide thick or thin-film ceramic sensors [125], yet, their production and development have progressed considerably, especially in laboratory studies [126]. Resistive elements [127,128] based on two main types of polyelectrolyte polymers [10] and copolymers [95], namely PMDS/PPDS and PSDA-b-PEG have been designed to detect humidity.

##### Ceramic Based Resistive Humidity Sensors

Electroceramic materials having single/polycrystalline structures, as single species or composites, could be great contenders for humidity sensing applications. The use of these innovative materials with novel humidity sensing properties is being investigated to overcome some of the limitations of many of the conventional materials, such as insufficient sensitivity or selectivity, low catalytic grade, insufficiency of cavities, surface degradation due to harsh contaminants in extreme conditions, and failure to operate in extremely dry or moistened environments. The addition of suitable nanomaterials with variable particle sizes/morphologies, hybridization of materials by replacement or doping of new atoms in the lattice, and particle size reduction to sub-nano scales can all be used to overcome most of the flaws.

With techniques such as thick film screen printing, porous ceramic or nanorod-based ceramic humidity sensors have also been manufactured and produced [129,130], whereby thin plasma or vacuum vapor films based on semiconducting metal oxides are formed onto an insulating substrate, conductive and nonconductive pastes are coated onto an insulating substrate [131,132], but also anodized films, which are typically used for aluminum oxide (Al_2_O_3_) [133]. In this kind of thick film product, dopant agents have been introduced as reaction catalysts to pre-react powders as part of the synthesis process to accelerate the dissociation of water molecules into functional groups containing hydrogen and hydroxyl ions. The film thickness is generally larger than 10 µm because of their semiconducting nature, thin films made from vacuum vapor or plasma sputter deposited on various types of substrates such as silicon will also act as resistive type devices, operating mostly on ionic-electronic conduction. The film resistivity is mostly reduced by the surface hydroxyl ions, which changes the impedance.

Ionic and electrical conduction sensors, as indicated previously, are two types of resistive sensors. Surface chemisorption and physisorption are used by ionic conduction types of humidity sensors to measure ambient air relative humidity, and they include MgCr_2_O_4_-TiO_2_ [134], ZnCr_2_O_4_-LiZnVO_4_ [135], TiO_2_-K_2_Ti_6_O_3_ [136], (A_l_, Fe)_2_O_4_-TiO_2_ [137], MgFe_2_O_4_ [5], ZnO, TiO_2_ [138], and some are of the nano nanoscale size. Nitta et al. developed a MgCr_2_O_4_-TiO_2_ porous ceramic humidity sensor for microwave oven applications [139]. This material was powered by water molecules’ chemisorption and physisorption, as well as ionic protonic conduction.

##### Perovskites and Perovskite-Type Ceramics-Based Resistive Humidity Sensors

The research and analysis of perovskite films and bulk materials’ humidity sensing behavior and morphological structure continue to generate unique preliminary and novel findings [140,141,142]. The humidity sensing process of perovskite-type materials with an empirical formula of ABX3 was reported to be based on electron transfer from water vapor molecules. Gas sensors have long employed perovskite-type materials with a composition of ABX_3_ (where X is a halogen or oxygen). [143], and for other applications as well. As can be seen from Table 1, some perovskite-type materials, such as PbTiO_3_ [97], ZnSnO_3_ [98], and NaTaO_3_ [99], were claimed to be utilized in the production of humidity sensors with fast reaction times, long-term stability, and prospective uses.

Zhang et al. [99] were able to hydrothermally synthesize NaTaO_3_ nanocrystalline films with great sensitivity, strong linearity with impedance values ranging in more than three times the normal magnitude, limited hysteresis, and quick response time at 100 Hz, AC 1 V from 33 to 95 percent relative humidity. The complex impedance was also used to investigate the humidity sensing mechanism. The findings suggested that NaTaO_3_ may be used in humidity sensors. In the example of ZnSnO_3_, Bauskar et al. [98] conducted research in which they were able to employ ZnSnO_3_ cubic crystallites produced using a hydrothermal technique to create a quick and stable humidity sensor. Linearity, rapid response and recovery behavior, hysteresis within 3.5 percent, outstanding repeatability, stability, and a wide range of operation (11–97% RH) were all excellent humidity sensing properties of the sensor. Finally, Mahmoud et al. [97] were able to employ PbTiO_3_ powder for moisture sensing measurements. The solid-state reaction approach was used to make perovskite PbTiO_3_ (PT) powder and ceramics in a cost-effective manner. It made it possible to create a perovskite tetragonal single phase. At an ambient temperature, however, pure PbTiO_3_ did not demonstrate a satisfactory reaction to relative humidity.

Hu et al. [144] developed a new type of humidity-sensitive device based on CH_3_NH_3_PbI_3-x_Cl_x_ films and investigated its sensitivity in RH ranges of 32 to 97 percent with a recovery time of 74 s. Xu et al. [145] have developed a CH_3_NH_3_PbBr_3_ humidity sensor with a quicker response time (250 s) and quicker recovery (30–70 s) in a broader range of humidity detection (7–98%). A CH_3_NH_3_PbI_3-x_Cl_x_-based humidity sensor with a reaction time of 21 s, quicker than the quickest commonly available psychrometer on the market, was recently produced by Ren et al. [100] with vertically oriented nanosheet arrays. Notwithstanding the significant efforts to enhance the performance of perovskite-based humidity sensors, humidity sensitive devices have proven to degrade over time due to the intrinsic chemical and phase instability of organic/inorganic perovskites, which is linked to low formation energy, high defect density, component separation, or phase separation [146,147,148,149]. Furthermore, the toxicity of lead remains a significant barrier to their practical adoption. In addition, structural variants devoid of Pb^2+^, such as double, triple and vacancy ordered perovskites, have gotten a lot of interest because of their flexibility in photoelectric devices. The produced metal-based halide perovskites such as (NH_4_)_3_Bi_2_I_9_ [150], Cs_2_AgBiBr_6_ [151], and Cs_3_Bi_2_Br_9_ [151], Cs_2_PdBr_6_ [37] and Cs_2_InBr_5_·H_2_O [39] provide intriguing potentials in a wide variety of optoelectronic applications due to the non-toxic, stability features of Bi^3+^_,_ Pd^2+^, In^+3^ with a comparable electron configuration to Pb^2+^.

#### 3.4.2. Capacitive Type Humidity Sensors

**Figure 6:**
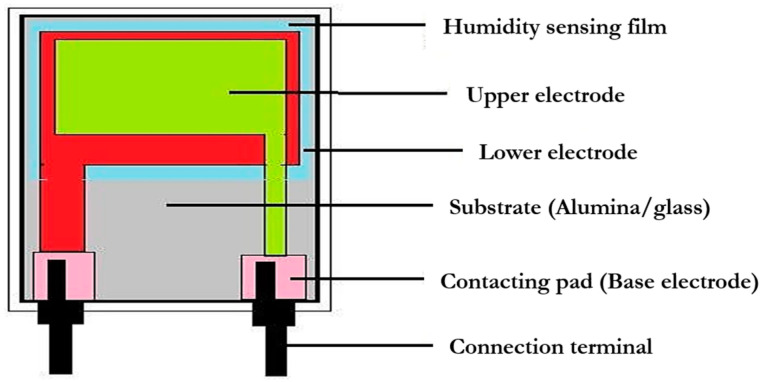
Configuration of a capacitive humidity sensor.

Capacitive humidity sensors are often designed with a layered structure with two electrode interfaces, or as an interdigitated structure with comb electrodes, analogous to resistive RH sensors, with the insulating polymer film sandwiched in between [101,127]. Several capacitive RH sensors have also been designed and fabricated employing this platform, which uses printing deposition or coating processes to deposit organic polymer thin films or porous ceramics such as alumina, perovskites, and porous silicon onto a ceramic substrate [152,153]. In a parallel plate setup, two metal electrodes are put on the substrate and coated with a thin film layer of a dielectric polymer or a porous ceramic metal oxide. As an upper electrode, a small coating of evaporated gold is applied to the top of the sensor surface to shield it from ambient pollution or dust and to assist in condensation. In a sandwich design, the top porous electrode is always a water vapor permeable layer [152]. Humicape, a capacitive-type thin film humidity sensor developed by Vaisala in Finland, has been extensively used in radiosonde applications and other humidity monitoring devices [101]. The sensor design is shown in Figure 6. 

##### Polymer-Based Capacitive Humidity Sensors

Polymeric capacitive humidity sensors have been widely employed in the industrial and automation industries due to their simplicity in terms of coating, mass production, long-term stability, and a wide variety of potential sensing polymers such as polyimides [154,155]. On-wafer silicon substrate parallel plate PI acid capacitor [156], capacitive sensors based on interdigitated electrodes and heating elements [157], high-sensitivity MEMS-based sensors [158], capacitive sensors that use doped ion-conducting polymers [159], and thin film-based cross-linked polyimide capacitive-type humidity sensors [144,160] are just a handful of good reports on capacitive sensors that have already been published.

Variations in the sensing film dielectric constant as a result of water absorption have a big impact on the capacitive RH sensors’ architecture, which alters the overall capacitance of the system. Differences in capacitance can be used to detect humidity changes because polymer dielectrics’ physical properties, such as permittivity, alter proportionally with the high dipole moments of water molecules. At ambient temperature, capacitive polymer sensors have a relative dielectric permittivity of roughly 5, whereas pure water has a permittivity of about 80. The dielectric permittivity increases the magnitude of the adsorption of water vapor by polymers (78.54), resulting in a sensitive linear change in capacitance. Polyimides (PI) and cellulose acetates are examples of such polymers, with relative dielectric permittivity values in the range from 3 to 6.

Based on established observations, the porosity of the humidity-sensitive polymer sheet may be changed to boost sensor responses. In 1983, Delapierre et al. [108] proposed the tensile-stressed fracture approach on thin films, which produces a large number of fractures in the film and appeared to be a useful strategy at the time without reducing conductivity or causing any damage. A porous chromium electrode was evaporated under conditions that caused the sensitive film to be tensile strained, resulting in the emergence of a significant number of fractures in the film, resulting in many orders of magnitude greater water vapor permeability rates. A small capacitive sensor composed of multi-wall carbon nanotubes (MWCNTs) may naturally generate porous nano-structures with greater sensing resolution, according to Yeow et al. [161]. The enhanced performance was attributed to the capillary condensation phenomenon. Dai et al. [10] investigated the responsiveness of polyelectrolyte humidity sensors in research. The humidity-sensitive polyelectrolyte on the substrate imprinted with interdigitated electrodes in situ was crosslinked using a thiol-ene click reaction process. Capabilities for high water adsorption and desorption, high stability, and repeatability were discovered in the polyelectrolyte humidity sensor. When altering humidity between 33 percent and 95 percent, the sensor demonstrated an ultrafast reaction and recovery (0.29 s/0.47 s), indicating that it might be used for breath monitoring and touchless sensing. 

Yang et al. [8] made a capacitive humidity sensor with excellent sensitivity built of nano fibrillated cellulose (NFC), graphene oxide (GO), and polydimethylsiloxane (PDMS) using a simple ultrasonic dispersion and freeze-drying procedure. NFC and GO with good water molecule adherence were used as substrates to improve the capacitive responsiveness of the humidity sensor. To avoid major fractures from occurring during the freeze-drying process, anhydrous ethanol was added to the humidity sensor, culminating in a regular network porous structure with a large number of conduction channels and active sites for molecular water. Furthermore, the use of PDMS increased the porous structure’s flexibility and stability. According to the findings, the humidity sensor with 30 wt% GO showed high humidity sensitivity (6576.41 pF/ percent RH), incredible repeatability, low humidity hysteresis characteristic in 11–97 percent relative humidity (RH) at 25 °C and short response/recovery times (57 s/2 s).

Bi et al. [93] developed a microscale capacitive humidity sensor that relied only on graphene oxide (G-O) sheets as humidity sensing materials. If compared to conventional capacitive humidity sensors, the G-O-based humidity sensor has a sensitivity of up to 37,800 percent, which is more than 10 times higher than the best conventional sensor for 15 percent to 95 percent relative humidity. This humidity sensor also has a fast reaction time (less than 1/4 of a standard one) and recovery time (less than 1/2 of a standard one). As a result, G-O has been identified as one of the best materials for manufacturing ultra-sensitive humidity sensors for a variety of applications. 

##### Ceramic-Based Capacitive Humidity Sensors

Aluminum oxide (Al_2_O_3_) with a microporous structure is the best material for these types of sensors [162]. Al_2_O_3_ with a small pore radius is extremely prone to very low humidity levels, according to the electron tunneling mechanism effect inside the compacted immobile layers of water [162]. In humidity sensing applications, only the two phases Al_2_O_3_ (amorphous) and Al_2_O_3_ (corundum) are often used; however, the former is somewhat more sensitive than the latter due to its higher porosity [27]. Anodization is a popular process in preparing porous Al_2_O_3_ humidity sensors for their low cost and convenience of usage. As per the electron tunneling mechanism effect within the condensed immobile layers of water, Al_2_O_3_ with a tiny pore radius is particularly sensitive to very low humidity levels. Aluminum oxide (Al_2_O_3_) with a porous structure is the most suited material for these types of sensors. Only the two phases γ-Al_2_O_3_ (amorphous) and α-Al_2_O_3_ (corundum) are typically utilized in humidity sensing applications; however, the former is more sensitive than the latter due to its greater porosity [27]. Because of its low cost and ease of use, anodization is a common step in the production of porous Al_2_O_3_ humidity sensors. McGhee et al. [6] created a humidity detecting device utilizing sputter-coated indium–tin oxide (ITO) and printed dielectric structures, which was tested for materials with sheet resistances ranging from 10 to 50 /sq. The ITO/Polymer composite sensors were utilized to develop a parallel-plate capacitive-based humidity sensor that can detect relative humidity in a test range of 5% to 95%. Such sensors were reported to be extremely dependable, having a linear response range of 5% to 75% relative humidity. The humidity sensors had an average reaction time of 31.5 s and a recovery time of 31 s in capacitive mode.

Both resistance and capacitance changes may very well be measured in these materials using a parallel plate or interdigitated structure, however, in this case, it is preferable to measure capacitance fluctuations. The majority of these ceramics are deposited as thick or thin films, then post-annealed to develop grains. Highly sensitive ZnO materials were employed in the fabrication of humidity sensors, where they were doped with LiCl. Wang et al. used carbon interdigital electrodes to screen-print pure ZnO nanofibers and LiCl-doped ZnO composite fibers on ceramic substrates [92]. High humidity sensitivity, quick reaction and recovery, minor hysteresis, great linearity, and good repeatability were all seen in the LiCl-doped sample’s findings. The sensor’s resistance increased by more than four orders of magnitude over the entire relative humidity (RH) range of 11 to 95 percent. The sensor’s reaction and recovery times were measured to be around 3 and 6 s, respectively. These findings demonstrated that this material might be a promising choice for making high-performance humidity sensors once again. 

On glass substrates, Gu et al. built ZnO/TiO_2_ core/shell nanorod capacitive thin-film humidity sensors. Sol-gel processed anatase titanium oxide (TiO_2_) shells were placed over hydrothermally generated zinc oxide (ZnO) nanorod cores. Per the morphological study, the initial zinc oxide nanorods were coated by anatase titanium oxide shells as a second layer. The ZnO/TiO_2_ nanocomposite (ZTNA) sensors have substantially better sensitivity at 95 percent RH than separate ZnO and TiO_2_ sensors (31 and 1380 times greater than the ZnO nanorod arrays and TiO_2_ thin films, respectively). Moreover, at ambient temperature, the capacitance of the core/shell arrays varied from 101 to 106 pF throughout the whole humidity range of 11 to 95 percent RH [163]. 

## 4. Conclusions and Future Perspectives

The design configurations of impedance- (resistive) and capacitive-based humidity sensors that have proven to be the best suited and most prevalent in advanced applications such as laboratory research or automated industries were examined. An interesting fact to note was that resistive sensors operate on the same principle as capacitive sensors, measuring electrical changes to provide a relative humidity value. Although resistive sensors use hygroscopic materials similar to capacitive sensors, the humidity measurement processes in these systems differ. The distinction made was that the resistance change in the material is measured instead of the capacitance, and capacitive measurement, which is based on electrical capacitance, is used by a capacitive humidity sensor. In comparison to resistive measurements, capacitive measurements are widely utilized in the literature because they are more resilient and stable with temperature and humidity changes. Although both capacitive and resistive sensors have the same goal, which is that of measuring humidity, their methodologies are different. Capacitive sensors have also proven to be more accurate and stable than resistive sensors while giving reproducible findings, making them more ideal for medical applications where precision is critical. However, they are more expensive than resistive sensors. The less expensive resistive sensors are more commonly utilized in circumstances where frequent measurements are required but precision is not required.

The success of these configurations moreover is linked to their ability to address common demands such as streamlined construction, free selection of sensing materials from several accessible kinds, cost, circuit adaptability, ease of manufacture, and measurement setup. Due to advantages such as low cost, proper gasketing, design flexibility, and quick deposition rate, film-based humidity sensors are more frequently employed. Semiconducting metal oxide and metal oxide/polymer-based sensors, which are primarily manufactured using thick film and thin film deposition techniques, stand out among the various types of humidity sensors due to their wide range of sensitive element options, post-processing capabilities, and superior response characteristics. 

Exploration of the synthesis and use of lead-free metal halide perovskites, such as Cs_2_BX_6_ or Cs_2_B”B’X_6_ and Cs_2_InX_5_.H_2_O, should be prioritized as a viable alternative to the endless difficulties that plagued other metal halides and oxides perovskites with lower stabilities and more efficient performances. Their nanoscale qualities may boost performance, having a significant impact on accuracy, reliability, and cost. In the future, nanocrystalline lead-free perovskite composites with ceramics, polymers, or ceramics/polymers, which are among the most promising materials for humidity sensors, may yield significant improvements in terms of humidity sensing performances.

## Figures and Tables

**Table 1:** Types of humidity sensors.

	Advantages	Disadvantages	Applications
Capacitive	Near-linear output voltage Long-term stability of results Helps to detect a wide range of RH	There is a short distance between the sensor and the signaling circuit.	Refrigerators, Ovens, and Dryers HVAC Systems Printers and Fax Machines Automobiles Food
Resistive	Low cost Small size There can be a significant distance between the sensor and the signal circuit. Highly interchangeable	Chemical vapors and other contaminants make them hypersensitive. When using water-soluble products, the output measurements may vary.	There is a variety of industrial, household, residential, as well as commercial uses.
Thermal conductivity	Appropriate for high-temperature environments and high corrosive situations Very durable When compared to other varieties, it has a higher resolution.	Any gas with differing thermal characteristics than Nitrogen may alter the reading measurement.	Pharmaceutical plants Ovens Clothes dryers and drying machines Food dehydration Drying kilns
